# Radiation and the Risk of Chronic Lymphocytic and Other Leukemias among Chornobyl Cleanup Workers

**DOI:** 10.1289/ehp.1204996

**Published:** 2012-11-08

**Authors:** Lydia B. Zablotska, Dimitry Bazyka, Jay H. Lubin, Nataliya Gudzenko, Mark P. Little, Maureen Hatch, Stuart Finch, Irina Dyagil, Robert F. Reiss, Vadim V. Chumak, Andre Bouville, Vladimir Drozdovitch, Victor P. Kryuchkov, Ivan Golovanov, Elena Bakhanova, Nataliya Babkina, Tatiana Lubarets, Volodymyr Bebeshko, Anatoly Romanenko, Kiyohiko Mabuchi

**Affiliations:** 1 Department of Epidemiology and Biostatistics, School of Medicine, University of California, San Francisco, San Francisco, California, USA; 2 National Research Center for Radiation Medicine, Kyiv, Ukraine; 3 Division of Cancer Epidemiology and Genetics, National Cancer Institute, National Institutes of Health, Department of Health and Human Services, Bethesda, Maryland, USA; 4 Robert Wood Johnson Medical School, Camden, New Jersey, USA; 5 Department of Pathology and Cell Biology, and; 6 Department of Medicine, College of Physicians and Surgeons, Columbia University, New York, New York, USA; 7 Burnasyan Federal Medical Biophysical Centre, Moscow, Russia

**Keywords:** Chernobyl nuclear accident, Chornobyl, Ukraine, chronic lymphocytic leukemia, leukemia, matched case–control study, radiation, radiation dose–response relationship, radiation-induced leukemia

## Abstract

Background: Risks of most types of leukemia from exposure to acute high doses of ionizing radiation are well known, but risks associated with protracted exposures, as well as associations between radiation and chronic lymphocytic leukemia (CLL), are not clear.

Objectives: We estimated relative risks of CLL and non-CLL from protracted exposures to low-dose ionizing radiation.

Methods: A nested case–control study was conducted in a cohort of 110,645 Ukrainian cleanup workers of the 1986 Chornobyl nuclear power plant accident. Cases of incident leukemia diagnosed in 1986–2006 were confirmed by a panel of expert hematologists/hematopathologists. Controls were matched to cases on place of residence and year of birth. We estimated individual bone marrow radiation doses by the Realistic Analytical Dose Reconstruction with Uncertainty Estimation (RADRUE) method. We then used a conditional logistic regression model to estimate excess relative risk of leukemia per gray (ERR/Gy) of radiation dose.

Results: We found a significant linear dose response for all leukemia [137 cases, ERR/Gy = 1.26 (95% CI: 0.03, 3.58]. There were nonsignificant positive dose responses for both CLL and non-CLL (ERR/Gy = 0.76 and 1.87, respectively). In our primary analysis excluding 20 cases with direct in-person interviews < 2 years from start of chemotherapy with an anomalous finding of ERR/Gy = –0.47 (95% CI: < –0.47, 1.02), the ERR/Gy for the remaining 117 cases was 2.38 (95% CI: 0.49, 5.87). For CLL, the ERR/Gy was 2.58 (95% CI: 0.02, 8.43), and for non-CLL, ERR/Gy was 2.21 (95% CI: 0.05, 7.61). Altogether, 16% of leukemia cases (18% of CLL, 15% of non-CLL) were attributed to radiation exposure.

Conclusions: Exposure to low doses and to low dose-rates of radiation from post-Chornobyl cleanup work was associated with a significant increase in risk of leukemia, which was statistically consistent with estimates for the Japanese atomic bomb survivors. Based on the primary analysis, we conclude that CLL and non-CLL are both radiosensitive.

It is well known that substantial risks of leukemia are associated with exposure to high acute doses of ionizing radiation [United Nations Scientific Committee on the Effects of Atomic Radiation (UNSCEAR 2008)]. Risks of leukemia associated with protracted exposures to low doses of radiation, which occur among occupationally exposed nuclear industry workers (Cardis et al. 2007; Muirhead et al. 2009) or among the general public living in areas affected by accidental releases of radioactive materials (Krestinina et al. 2010), have been reported to be of similar magnitude, but several questions remain (Jacob et al. 2009; Richardson 2009; UNSCEAR 2010). Of special concern are radiation-related leukemia risks among those who are engaged in emergency and recovery work after nuclear facility accidents because the level of exposure can be relatively high. As of 2006, over 500,000 persons from Belarus, the Russian Federation, and Ukraine had been registered as emergency and recovery workers after the 1986 Chornobyl accident (UNSCEAR 2011).

Although most types of leukemia are known to be radiogenic (Little et al. 1999; Preston et al. 1994), to date very few studies have provided substantial evidence for a radiogenic excess of chronic lymphocytic leukemia (CLL) (UNSCEAR 2008). However, the view that CLL is not caused by radiation has been questioned (Linet et al. 2007; Richardson et al. 2005), and more recent studies based on incident rather than mortality outcomes have suggested a radiation effect on CLL as well as on other types of leukemia (Kesminiene et al. 2008; Lane et al. 2010; Mohner et al. 2010; Rericha et al. 2006; Romanenko et al. 2008b).

In our previous study of leukemia occurring between 1986 and 2000 among Chornobyl cleanup workers from Ukraine (Romanenko et al. 2008b), we found a significantly increased risk of leukemia, similar in magnitude to the estimate from the Japanese atomic bomb survivors (UNSCEAR 2008). The data indicated elevated risks for both CLL and other leukemias. We therefore extended the study through 2006, with a near doubling of the number of leukemia cases. We herein report results of the analysis of the extended data.

## Methods

*Study data.* Data were from a nested case–control study in a cohort of 110,645 male Ukrainian workers who were 20–60 years of age during cleanup activities in 1986–1990 after the Chornobyl nuclear power plant accident and who were registered in the Chornobyl State Registry of Ukraine (SRU) before 1992 and resided in Kyiv City or in any one of five study oblasts (areas similar to a state or province: Cherkasy, Chernihiv, Dnipropetrovsk, Kharkiv, and Kyiv) at the time of registration (Romanenko et al. 2008a).

Potential cases for the period of 1986–2000 were identified among persons diagnosed with leukemia or with a diagnosis from a broad screening list of 99 ancillary conditions that might possibly represent cases of leukemia (including myelodysplasia, non-Hodgkin lymphoma, and multiple myeloma) at all health care institutions in the study area; potential cases were then used to create a Provisional Leukemia Registry (Romanenko et al. 2008a). Potential cases during 2001–2006 were identified by linkage of the SRU cohort with the Ukrainian Cancer Registry (UCR), which achieved nationwide coverage in 1997 (Fedorenko et al. 2011).

A total of 162 cases of leukemia were confirmed by the International Hematology Panel of five hematologists/hematopathologists. Most cases were confirmed unanimously after initial review of the cytological material and medical records or, lacking such initial unanimity, by a mutually acceptable consensus diagnosis after reexamination of all materials and in-depth discussion between the panel members. Descriptions of the clinical courses and histological confirmation of the diagnoses from the medical records were available for all cases. Bone marrow aspirates/biopsy slides and/or peripheral blood smears were available for 113 cases (70%). Acute leukemia types were classified using the World Health Organization system of classification (Jaffe et al. 2001). CLL diagnoses were based on the criteria established by the U.S. National Cancer Institute (NCI) Working Group (Cheson et al. 1996). The diagnostic confirmation rate for CLL (89%) and non-CLL cases (79%) did not differ significantly (*p* = 0.103).

With a targeted 5:1 control:case ratio, we used incidence–density sampling to randomly select 5–9 controls for each potential case from members of the cohort who were alive and at risk at the time of the case diagnosis and were matched to the case on place of residence (in one of five oblasts or Kyiv City) and year of birth, regardless of whether the potential control was alive at the time of ascertainment. Among 1,364 selected controls, 901 were interviewed, 215 refused to participate, 213 could not be traced, and 35 moved out of the study regions. Response rates, including untraceable subjects, were 70% for live controls, 49% for next-of-kin, and 64% for colleagues responding for deceased controls. There were 677 controls interviewed for 137 confirmed and interviewed leukemia cases. In addition, 224 controls were interviewed for cases that were not subsequently interviewed (directly or by proxy) or not confirmed. We rematched 186 of the latter controls to confirmed cases using the matching criteria, resulting in a total of 863 controls. We used all 863 controls in the analyses because results with and without the extra controls were similar (data not shown).

A time-and-motion dose reconstruction method, known as Realistic Analytical Dose Reconstruction with Uncertainty Estimation (RADRUE), was developed specifically for this study and for a similar study conducted in Belarus, Russia, and Baltic countries (Kesminiene et al. 2008) by an international group of scientists including experts from Belarus, France, Russia, the United States, and Ukraine (Chumak et al. 2008; Kryuchkov et al. 2009). The method used combined data on work history from dosimetric questionnaires with field radioactivity measurements to estimate individual bone marrow doses for all study subjects. In-person interviews were conducted by trained interviewers and included questions concerning locations of work and residence while in the 30-km exclusion zone around the Chornobyl nuclear power plant, types of work, transportation routes, and corresponding dates. For deceased cases or controls, proxy interviews were conducted with next-of-kin for demographic and medical information and with co-workers for work histories in the 30-km exclusion zone. Proxy interviews were conducted for 69 deceased cases (38 non-CLL and 31 CLL, 50% of all cases) and 43 deceased controls (5% of all controls).

Radiation dose estimates were not available for 25 cases (15%): 2 were ineligible, 17 could not be traced, 4 refused to complete the dosimetry questionnaire, and 2 had poor quality of interview response. Response rates were 96% for live cases and 79% for next-of-kin and colleagues responding for deceased cases. The present study thus included 137 confirmed cases with radiation dose estimates, 79 CLL and 58 non-CLL cases [6 with acute lymphocytic leukemia, 16 with acute myeloid leukemia, 7 with acute leukemia/not otherwise specified, 24 chronic myeloid leukemia, and 5 with other chronic leukemia (2 large granular lymphocyte leukemia–natural killer cell type, and 3 large granular lymphocyte leukemia–T-cell type)].

The protocol for the study was approved by the institutional review boards of the NCI (Bethesda, MD, USA); the University of California, San Francisco, School of Medicine (San Francisco, CA, USA); and the National Research Center for Radiation Medicine (Kyiv, Ukraine). All participants gave written informed consent.

*Statistical analysis.* As in our previous study (Romanenko et al. 2008b), we fitted a conditional logistic regression model that assumed a linear dose–response relationship although we evaluated several alternative forms, including linear–quadratic, exponential, and power models. The model was fitted by maximum likelihood (McCullagh and Nelder 1989) using the EPICURE statistical package (Preston et al. 1993). The excess relative risk per gray (ERR/Gy) computed by this model is an estimate of the excess risk associated with exposure to 1 Gy relative to no radiation exposure. We also estimated relative risks (RRs) for radiation dose categories. Using likelihood ratio tests, we examined the potential modifications of association between radiation and the disease outcomes by means of interaction terms between radiation dose (continuous) and indicator terms for categorical variables (leukemia subtype, proxy status, 0–1 vs. 2–15 years from start of chemotherapy to direct interview, and type of work performed in the 30-km Chornobyl zone) or continuous variables (year of case diagnosis, time since first exposure, and age at first exposure), although for ease of presentation, the ERR/Gy estimates are shown for categories of continuous variables. The population-attributable risks (PARs) of all leukemia, CLL, and non-CLL were estimated as the reduction in the leukemia risk after elimination of radiation exposure as a fraction of the total leukemia risk:

PAR = Σ*_k_* P*_k_* × (RR*_k_* – 1)/Σ*_k_* P*_k_* × RR*_k_*, [1]

where *k* = 0, 1, …, 100, and P*_k_* and RR*_k_* are the proportion and model-based estimates of RR at the *k*th percentile dose level. For these computations, we approximated the bone marrow dose distribution by using percentiles. Confidence limits for PAR were based on the substitution method (Daly 1998).

Our analyses were based on the cumulative doses derived as the sums of the arithmetic means of the annual 1986–1990 bone marrow doses estimated by generating 10,000 realizations of dose predictions from the RADRUE method (Chumak et al. 2008). We assessed lag interval, a period of recent exposure assumed unrelated to disease, for the calculation of cumulative dose from 1986 to 1990 in 1-year increments between 0 and 10 years. The deviance, a measure of model fit, was minimized for both CLL and non-CLL analyses when we set the lag interval to either 1 or 2 years [see Supplemental Material, Table S1 (http://dx.doi.org/10.1289/ehp.1204996)], although the deviances were very similar for up to a lag of 5 years. When 20 cases who were interviewed < 2 years from start of chemotherapy were excluded, the optimal lag both for CLL and non-CLL was 2 years. Choice of lag had little effect on the risk estimates (results not shown). Since various other bodies (Committee to Assess Health Risks from Exposure to Low Levels of Ionizing Radiation 2006; UNSCEAR 2008) recommend a lag of 2 years for non-CLL, we lagged radiation doses by 2 years in all analyses.

Tests of all hypotheses were based on likelihood ratio tests. All tests were two-sided with a specified type I error of 0.05 and confidence intervals (CIs) for risk estimates were derived by the profile likelihood method (McCullagh and Nelder 1989). If the likelihood being sought for a lower bound estimate did not converge, it was given by < –1/D_max_, where D_max_ was the maximum radiation dose.

## Results

The age at diagnosis of 137 cases ranged from 25 to 78 years (median, 56) and the corresponding age for 863 controls ranged from 25 to 79 years (median, 55). Mean ± SD estimated bone marrow radiation doses for cases and controls were 132.3 ± 342.6 mGy and 81.8 ± 193.7 mGy, respectively (Table 1). Seventy-eight percent of study participants had bone marrow doses < 100 mGy, and 87% < 200 mGy. Cases and controls did not differ significantly by urban versus rural residential status at the time of interview, age at first radiation exposure in the 30-km Chornobyl zone, or education; however, more cases than controls were proxy-interviewed (*p* < 0.001) (Table 1). Cases and controls did not differ significantly by calendar year of first cleanup mission, type of work or total number of missions, or by self-reported smoking, alcohol consumption, medical or diagnostic radiation exposures, or occupational exposures to chemicals or radiation before and after the Chornobyl accident (results not shown). Thirty-eight percent of cleanup workers were in the 30-km zone around the Chornobyl nuclear power plant for > 2 months (median time in the zone for all workers, 35 days; range, 1–1,711 days; similar for cases and controls, *p* Wilcoxon = 0.729).

**Table 1:** Descriptive characteristics [*n* (%)] of cases and controls identified during follow-up (1986–2006).

Characteristic	Cases (n = 137)	Controls (n = 863)	p-Value^a^
Radiation dose, mGy [mean ± SD (range)]b	132.3 ± 342.6 (0–3220.0)	81.8 ± 193.7 (0–2600.0)	0.119c
Year of birth			0.988
1923–1929	10 (7)	67 (8)
1930–1939	38 (28)	222 (26)
1940–1949	43 (31)	285 (33)
1950–1959	37 (27)	234 (27)
1960–1965	9 (7)	55 (6)
Areas of study			0.938
Cherkasy Oblast	7 (5)	60 (7)
Chernihiv Oblast	11 (8)	77 (9)
Dnipropetrovsk Oblast	26 (19)	155 (18)
Kharkiv Oblast	17 (12)	107 (12)
Kyiv Oblast	27 (20)	183 (21)
Kyiv City	49 (36)	281 (33)
Type of residence at time of interview			0.090
Urban	101 (74)	680 (79)
Rural	19 (14)	151 (18)
Other	10 (7)	32 (4)
Unknown	7 (5)	0 (0)
Age at first exposure (years)			0.970
20–34	31 (23)	207 (24)
35–41	36 (26)	221 (26)
42–49	40 (29)	239 (28)
50–63	30 (22)	196 (23)
Education			0.474
≤ 8 years	16 (12)	131 (15)
High school	46 (34)	341 (40)
Trade school	34 (25)	200 (23)
College	34 (25)	188 (22)
Unknown	7 (5)	3 (0)
Proxy interviews			< 0.001
No	68 (50)	820 (95)
Yes	69 (50)	43 (5)
ap-Value from the chi-square test unless otherwise stated. bBone marrow radiation dose lagged by 2 years. cp-Value from the Wilcoxon rank sum test.						

For all leukemias, we found a significant positive association with continuous radiation dose with an estimated ERR/Gy = 1.26 (95% CI: 0.03, 3.58, *p* = 0.041) (Table 2). However, preliminary analysis identified a significant (*p* = 0.021) difference in the dose response for 20 cases (6 non-CLL and 14 CLL) with direct in-person interviews < 2 years from start of chemotherapy compared with other cases [ERR/Gy = –0.47 (95% CI: < –0.47, 1.02, *p* = 0.244) for 20 cases vs. ERR/Gy = 2.38 (95% CI: 0.49, 5.87, *p* = 0.004) for the remaining 117 cases] [Table 2 and also see Supplemental Material, Table S2, (http://dx.doi.org/10.1289/ehp.1204996)]. Because of this marked disparity, we limited our primary analyses to cases who were interviewed 2–15 years after start of chemotherapy, did not have chemotherapy, or for whom proxy interviews were used and their matched controls (85% of all cases and 83% of all controls).

**Table 2:** ERR/Gy (95% CIs) for leukemia within categories of various factors.

Model description	Cases (n)	ERR/Gy (95% CI)	p-Value^a^	p Interaction^b^
All cases	137	1.26 (0.03, 3.58)	0.041
Excluding cases with direct interviews < 2 years from start of chemotherapy	117	2.38 (0.49, 5.87)	0.004
Leukemia subtype								
Non-CLL	52	2.21 (0.05, 7.61)	0.039	0.888
CLL	65	2.58 (0.02, 8.43)	0.047
Proxy statusc
Proxy	69	3.98 (< –0.15, 25.23)		0.420
Direct interview	48	0.88 (< –0.38, 5.28)
Year of case diagnosis
1986–1994	33	6.70 (0.27, 27.10)		0.141d
1995–2000	36	2.69 (–0.04, 11.23)
2001–2006	48	1.25 (< –0.69, 5.35)
Type of work performed in the 30-km Chornobyl zone								
Early responders	32	1.49 (–0.02, 5.07)		0.711
Military personnel	43	4.23 (0.12, 12.59)
Professional nuclear power workers	5	2.72 (< –0.91, 19.58)
Other	37	4.23 (–0.27, 15.25)
Time since first exposure (years)								
≤ 9	38	5.10 (–0.02, 19.17)		0.162d
10–14	34	4.09 (0.39, 13.47)
15–20	45	0.84 (< –0.78, 4.50)
Age at first exposure (years)								
20–34	27	1.01 (< –0.98, 8.65)		0.249d
35–41	30	1.61 (–0.49, 8.80)
42–49	33	5.67 (0.58, 21.79)
50–63	27	2.00 (< –0.38, 10.11)
Cases with direct interviews < 2 years from start of chemotherapy are excluded from all analyses except the “all cases” analysis. ap-Value of departure of ERR/Gy from zero. bp-Value for interaction effects. cBackground rate adjusted for proxy status. dp-Value from the linear trend test.								

**Figure 1:**
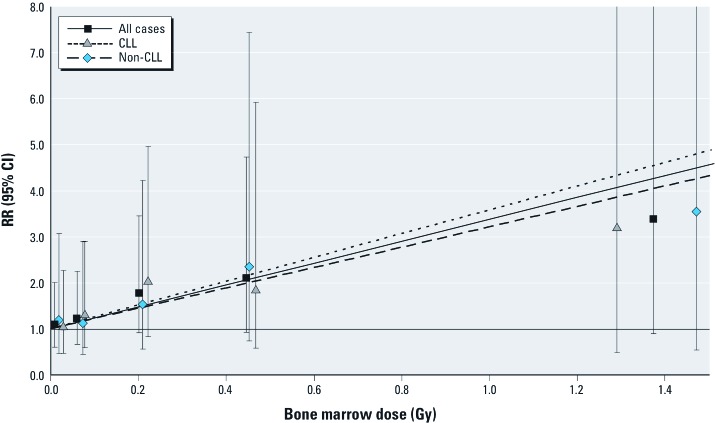
RRs (95% CIs) of leukemia by categories of radiation dose and fitted linear dose–response models. For display purposes, we added offsets to category mean doses on the abscissa coordinate to separate the overlapping estimates (10 mGy for non-CLL and 20 mGy for CLL analyses, respectively).

RRs increased with increasing radiation dose for all leukemia (Figure 1). Tests for quadratic, exponential, or power deviations from the linear dose response shown in Figure 1 were not significant (*p* = 0.927, *p* = 0.917, *p* = 0.267, respectively). The dose responses increased significantly for both non-CLL [ERR/Gy = 2.21 (95% CI: 0.05, 7.61, *p* = 0.039)] and CLL [ERR/Gy = 2.58 (95% CI: 0.02, 8.43, *p* = 0.047)] subtypes, with tests for interaction consistent with homogeneity (*p* = 0.888)] (Table 2).

We found no significant difference in ERR/Gy estimates by proxy or direct interviews (*p* = 0.420), calendar period of diagnosis (*p* = 0.141), or type of work performed in the 30-km Chornobyl zone (*p* = 0.711) (Table 2). Although also not significant, ERR/Gy estimates tended to decrease with increasing time (years) from first radiation exposure in the Chornobyl zone and to increase with increasing age at first exposure (*p* = 0.162, *p* = 0.249, respectively) (Table 2). The proportion of proxy versus direct interviews decreased over time (60.0%, 73.9%, 55.6%, and 54.2% for cases diagnosed in 1986–1989, 1990–1994, 1995–2000, and 2001–2006, respectively).

**Figure 2:**
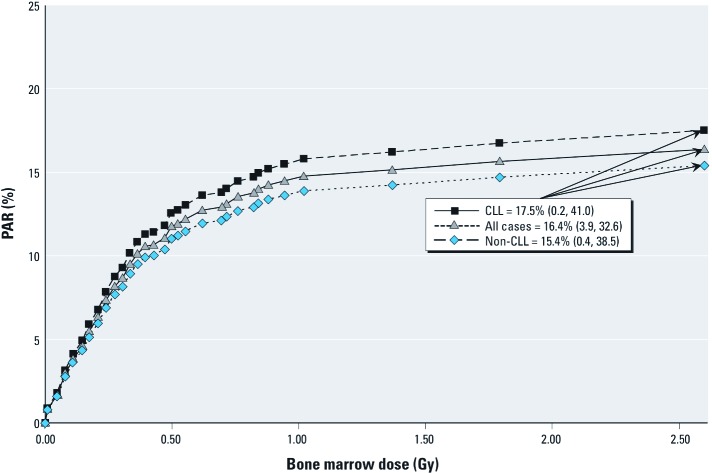
PARs of all leukemia and CLL and non-CLL, separately.

We estimated that approximately 16% of all leukemia cases in our Chornobyl cleanup worker population over a period of 20 years of follow-up [PAR = 16.4% (95% CI: 3.9, 32.6)] were attributable to radiation exposure from the Chornobyl accident. The majority of the PAR arose from dose groups of < 200 mGy in which there were large numbers of cleanup workers (Figure 2). Proportions of CLL and non-CLL cases attributable to radiation were similar, with PARs of 17.5% (95% CI: 0.2, 41.0) and 15.4% (95% CI: 0.4, 38.5), respectively.

For completeness, we evaluated modifications of the ERR/Gy presented in Table 2 using all case and control data [see Supplemental Material, Table S2 (http://dx.doi.org/10.1289/ehp.1204996)]. In general, results using the full dataset were consistent with the primary analysis. However, the ERR/Gy for CLL [0.76 (95% CI: < –0.38, 3.84, *p* = 0.352)] was lower than the estimated ERR/Gy for CLL from our primary analysis excluding 14 CLL cases [2.58, (95% CI: 0.02, 8.43, *p* = 0.047)]. In the analysis using the full dataset, as in the primary analysis, the ERRs were not significantly different between CLL and non-CLL outcomes (*p* = 0.536).

## Discussion

Here we report several important findings concerning the late effects of ionizing radiation exposure. First, our results confirm and significantly strengthen the evidence from our previous study (Romanenko et al. 2008b) that showed significant associations between protracted radiation exposure at low doses and leukemia incidence. Increased risks of leukemia, although not statistically significant, were also reported from a study of Chornobyl cleanup workers from Belarus, Russia, and Baltic countries (Kesminiene et al. 2008). Second, our results indicate that radiation risk estimates are elevated for both CLL and non-CLL. Generally, assessment of radiation risks of cancer and leukemia from exposures to low or protracted radiation doses derives from extrapolation of risks from epidemiological studies of populations exposed to single or high doses (e.g., studies of Japanese atomic bomb survivors and of medically exposed persons) (UNSCEAR 2008). It has been assumed that protraction of radiation dose results in a reduction of adverse biological effects, and an important uncertainty involved in these extrapolations relates to the risk associated with acute versus protracted exposure. The mean cumulative radiation doses (0.092 Gy) received by the Chornobyl cleanup workers were lower than reported for the atomic bomb survivors (0.24 Gy) (UNSCEAR 2008), and the ERR/Gy estimate of 2.21 (95% CI: 0.05, 7.61) for non-CLL was lower than the ERR/Gy of 3.98 (90% CI: 2.32, 6.45) for exposure at ≥ 40 years of age that can be estimated from the atomic bomb survivor data, although the estimates are comparable given the range of statistical uncertainty.

Chornobyl cleanup workers had higher radiation doses than those reported in other studies of incident leukemia after protracted radiation exposures, for example, the United Kingdom (mean = 0.025 Gy; Muirhead et al. 2009) or Canadian (0.007 Sv; Sont et al. 2001) radiation workers, Eldorado (0.052 Sv; Lane et al. 2010) or East German (0.024 Gy; Mohner et al. 2010) uranium miners, and the RRs of non-CLL leukemia were generally comparable [ERR/Gy = 1.78 (90% CI: 0.17, 4.36) for UK and ERR/Gy = 2.7 (90% CI: < 0, 18.8) for Canadian radiation workers]. Radiation-related risks of incident leukemia in the cohort of Techa River residents exposed to radioactive releases from the Mayak nuclear facility were higher but statistically comparable to the risks estimated in our study [ERR/Gy = 4.9 (95% CI: 1.6, 14.3) (Krestinina et al. 2010)], possibly related to the fact that 92% of their bone marrow dose (mean = 0.30 Gy) was due to internal exposures to strontium.

We estimated similar radiation-related risks for CLL and non-CLL in our primary analysis after excluding a subset of cases with interviews < 2 years from start of chemotherapy. The associations were attenuated when all cases were included in the analysis, particularly for CLL, but the ERRs for CLL and non-CLL were not significantly different in either analysis. The majority of epidemiological studies of radiation-exposed populations, whether from occupational or environmental exposures (Cardis et al. 2007; UNSCEAR 2008), or from therapeutic exposures (Boice et al. 1987; Curtis et al. 1994; Damber et al. 1995) have reported no excess of CLL. In reviewing the epidemiology and etiology of CLL, Linet et al. (2007) and Richardson et al. (2005) stressed the need for special care to ascertain CLL cases, especially when relying on information from death certificates, because of the dormant characteristics of this type of leukemia. It is thus pertinent that the recently emerging evidence of a radiogenic etiology for CLL derives mainly from incidence studies. In particular, indications for increased risks of CLL from radiation exposure have come from incidence studies of Chornobyl cleanup workers from Belarus, Russia, and Baltic countries (Kesminiene et al. 2008), and from uranium miners with exposures to alpha particles and gamma radiation in Canada, Germany, and Czechoslovakia (Lane et al. 2010; Mohner et al. 2010; Rericha et al. 2006). On the other hand, radiation and CLL were not associated according to analyses of incidence data in UK radiation workers (Muirhead et al. 2009) or the Techa River residents (Krestinina et al. 2010). The inconsistent results from studies of various exposed groups are puzzling, possibly implying diagnostic variability between the studies, and indicate the need for more intensive investigations in these and other irradiated populations.

While B cell–derived CLL may differ from other types of leukemia in etiology and pathogenesis, there is biological plausibility for the radiogenic potential for CLL. Mature B-cell CLLs are clonal proliferations of B cells at various stages of differentiation, and the initiating genetic lesions can occur in immature bone marrow B cells (Chiorazzi et al. 2005). Recent studies reported marked similarities in somatic mutations of CLL and other leukemias (Richardson et al. 2005). Also, it is possible that radiation may trigger the progression of benign monoclonal B-cell lymphocytosis, a putative precursor to CLL (Linet et al. 2007).

The strengths of this study include the large number of cases compared to studies of high- and moderate-dose exposures and of low-dose exposures among occupationally exposed workers, the selection of cases and controls from within a large cohort of cleanup workers of the 1986 Chornobyl nuclear power plant accident from Ukraine, the wide and rigorous search for diagnoses of leukemia, and the confirmation of all diagnoses by a panel of hematologists and hematopathologists based on medical records that were available for all cases, and biological materials (including bone marrow aspirates/biopsy slides and/or peripheral blood smears) that were available for 113 cases (70%). In particular, the diagnostic confirmation rates for CLL (89%) and non-CLL cases (79%) were high and comparable. In a study of cleanup workers from Belarus, Russia, and Baltic countries (Kesminiene et al. 2008), slides and case notes were available for review for 73% of cases, but 15% of the material submitted for review was judged to be inadequate for diagnosis. The interview participation rates in our study for both cases and controls as well as for alive subjects and proxies for deceased study subjects were reasonable. To minimize potential biases, interviewers were not aware of case–control status and were carefully trained not to ask probing questions beyond those listed on the questionnaire. Similarly, doses were estimated without knowledge of case–control status and members of the hematology panel did not know the radiation dose of cases under review. Finally, the information collected during interviews allowed us to estimate the effects of a number of potential confounders not generally available in other studies of cleanup workers (Ivanov 2007).

As in many retrospective case–control studies, recall bias can lead to biased estimation of radiation doses and is a concern in the present study. However, repeat interviews of alive subjects suggested good recall of missions within the Chornobyl cleanup zone (Kryuchkov et al. 2009). Fifty percent of case information was provided by proxy interviews. Mean bone marrow doses for subjects with direct and proxy interviews were not significantly different (*p* Wilcoxon = 0.577 and 0.512 for cases and controls, respectively). ERR/Gy estimates were higher, although not significantly so (*p* = 0.420), for proxy-interviewed than directly-interviewed subjects. Cleanup workers generally worked in groups and performed similar work, with co-worker proxies having first-hand knowledge about cleanup activities of deceased workers. Comparison of data from proxy interviews of live subjects with that from the subjects themselves resulted in comparable radiation dose estimates averaged over 102 pairs of subjects and proxies (geometric mean of the ratio of doses = 0.91), but large variabilities were suggested when ratios of doses for individual pairs of subjects and proxies were considered (Kryuchkov et al. 2009). Participation rates were higher for alive cases than for alive controls. Kesminiene et al. (2008) reported generally similar findings, with participation rates for cases also tending to be somewhat greater than for controls at 97% and 96%, respectively, for cleanup workers from Belarus; 87% and 91%, respectively, from Russia; and 82% and 73%, respectively, from Baltic countries.

Case ascertainment procedures varied during the study period of 1986–2006. As noted in the “Methods,” we identified cases using local health care facilities before 2001, whereas later cases were identified through the linkage of the cohort file with the UCR files. We compared ascertainment methods by using both procedures in Kyiv City. Case identification was identical, except for one recently diagnosed case that would have been reported to the UCR later in the year. In addition, we searched UCR files for cases diagnosed in 1986–2000 in areas other than study areas and did not identify any new cases among cohort members.

We observed a significant increase in the risk of leukemia with radiation dose based on the entire study sample. However, a preliminary examination of differences in various characteristics of participating cases, ascertained using the two methods described above, indicated that cases with direct in-person interviews < 2 years from start of chemotherapy treatment had mean bone marrow radiation dose estimates significantly lower than other cases interviewed in-person (16.8 vs. 121.4 mGy, 7-fold difference in means, *p* Wilcoxon = 0.036), and these doses were uniformly lower across all types of work performed in the 30-km zone, whereas the mean doses for controls from both groups were almost identical. The ERR/Gy estimates for cases with direct interviews < 2 years from start of chemotherapy (ERR/Gy = –0.47) and the remaining cases (ERR/Gy = 2.38) differed significantly (*p* = 0.021), with the former estimate incompatible with our current understanding of radiation-related leukemia risk. ERR/Gy estimates in the former group were negative overall and by time since first exposure, for cases diagnosed in 1986–2000 and 2001–2006, and for CLL and non-CLL cases (data not shown). The discrepancy could have arisen by chance or from an unknown ascertainment anomaly. Other possible reasons were that the 20 cases were undergoing therapy at the time of interview or were in poorer health compared to other cases, which could have influenced the accuracy of recall. In our primary analyses, we omitted these 20 cases so that results were not unduly influenced. Nevertheless, patterns of results using all cases were generally similar. In the analysis using all cases, the risks both for CLL and non-CLL were lower, particularly for CLL [0.76 (95% CI: < –0.38, 3.84, *p* = 0.352) vs. 2.58 (95% CI: 0.02, 8.43, *p* = 0.047)] [Table 2 and also see Supplemental Material, Table S2 (http://dx.doi.org/10.1289/ehp.1204996)]. In other respects, in relation to the variation of risks by year of case diagnosis, type of work performed, time since first exposure, or age at first exposure, the patterns were broadly similar (see Supplemental Material, Table S2). However, it must be recognized that our final results derived from a post hoc subgroup analysis.

The mean radiation doses for cases ascertained in 1986–2000 (Romanenko et al. 2008b) and 2001–2006 after excluding cases with direct in-person interviews < 2 years from start of chemotherapy treatment, were similar (mean ± SD) (143.8 ± 408.8 mGy and 152.0 ± 286.8, respectively, *p* Wilcoxon = 0.616), and there was no statistically significant difference in the dose response [ERR/Gy = 3.44 (95% CI: 0.47, 9.78) vs. ERR/Gy = 1.25 (95% CI: < –0.69, 5.35), *p* for interaction = 0.403, not shown]. Tests of linear trend for modifying effects of calendar year of diagnosis and years since first exposure were not statistically significant (*p* = 0.141 and *p* = 0.162, respectively, Table 2), but estimated radiation-related RRs of all leukemia generally tended to decrease. The decreasing temporal trend may have, at least partially, been due to the higher ERR/Gy associated with proxy interviews, which were conducted with many of the leukemia cases diagnosed in the early years after the accident.

The proportion of CLL cases in our study (58%) was higher than the approximately 40% reported by most population-based cancer registries (Dores et al. 2007) and 44% of all diagnosed leukemias among males in Ukraine (Gluzman et al. 2006). [Note that this number differs from the 29.32% reported in Gluzman et al. (2006), which was calculated as a proportion of CLL among all hematological malignancies, including multiple myeloma and NHL.] An earlier study suggested that cancer registries may be missing as much as 38% of CLL compared with the incidence of CLL detected using sophisticated measures such as flow cytometric immunophenotypic analysis (Zent et al. 2001). Using the age-specific incidence rate of CLL among men in Ukraine for 2003, we estimated that the number of CLL cases diagnosed in our cohort of 110,645 male cleanup workers over the period of 20 years after the accident was 60% higher than what would be expected for the general male population of Ukraine [standardized incidence ratio = 1.60 (95% CI: 1.3, 2.0)]. Although part of this increase could be due to estimated radiation effects of CLL, we speculate that performance of recommended annual medical examinations, including blood tests and a visit to a hematologist, for Chornobyl cleanup workers could have resulted in better case ascertainment and/or detection of cases at earlier stages than in a general population (Gluzman et al. 2006).

## Conclusions

Our findings provide important evidence of increased risk of leukemia associated with chronic protracted exposure to low doses of ionizing radiation. The finding from our primary analysis of similar radiogenic risks both for CLL and non-CLL was based on a well-defined population-based cohort, rigorous case ascertainment, and expert hematological review coupled with well-characterized radiation dose estimates. In our cohort of cleanup workers from 1986 through 2006, about 16% (19 cases) of all leukemia were attributed to radiation exposure, with similar estimates for non-CLL (15%) and CLL (18%). CLL is the most common type of leukemia in this cleanup worker population and, as the workers age, CLL cases will rapidly increase, raising concerns for medical consequences. The radiogenic risk for CLL also has important public health implications in other populations because it is the most prevalent type of leukemia in Western populations, with approximately 16,000 cases estimated to be diagnosed in the United States in 2012 (Howlader et al. 2012). Further investigations are needed to develop a better understanding of the association between radiation and CLL.

## Supplemental Material

(12 KB) PDF
