# Supplementary material for: Radiation and the Risk of Chronic Lymphocytic and Other Leukemias among Chornobyl Cleanup Workers
Source: Environ Health Perspect. 2012 Nov 8;121(1):59–65. doi: 10.1289/ehp.1204996 (PMC3553431; doi:10.1289/ehp.1204996)
Supplement: (12 KB) PDF [file ehp.1204996.s001.pdf]

Supplemental Material

Radiation and the Risk of Chronic Lymphocytic and Other Leukemias among Chornobyl Cleanup Workers

Lydia B. Zablotska,[1] Dimitry Bazyka,[2] Jay H. Lubin,[3] Nataliya Gudzenko,[2] Mark P. Little,[3] Maureen Hatch,[3] Stuart Finch,[4] Irina Dyagil,[2] Robert F. Reiss,[5] Vadim V. Chumak,[2] Andre Bouville,[3] Vladimir Drozdovitch,[3] Victor P. Kryuchkov,[6] Ivan Golovanov,[6] Elena Bakhanova,[2] Nataliya Babkina,[2] Tatiana Lubarets,[2] Volodymyr Bebeshko,[2] Anatoly Romanenko,[2] Kiyohiko Mabuchi[3]

[1] Department of Epidemiology and Biostatistics, School of Medicine, University of California San Francisco, San Francisco, California, USA

[2] National Research Center for Radiation Medicine, Kyiv, Ukraine

[3] Division of Cancer Epidemiology and Genetics, National Cancer Institute, National Institutes of Health, Department of Health and Human Services, Bethesda, Maryland, USA

[4] Robert Wood Johnson Medical School, Camden, New Jersey, USA

[5] Departments of Pathology and Medicine, College of Physicians and Surgeons, Columbia University, New York, New York, USA

[6] Burnasyan Federal Medical Biophysical Centre, Moscow, Russia

Supplemental Material, Table S1: Comparison of deviances from analyses using various lag times.

	Latent period (years)					
	**1**	**2**	**3**	**4**	**5**	**10**
All cases without exclusions (n=137)						
All cases	525.5	525.6	526.0	526.2	526.0	527.9
CLL	301.4	301.4	301.6	301.8	301.8	302.3
non-CLL	223.6	223.8	223.9	223.7	223.3	223.5
Excluding cases with direct interviews less than 2 years from start of chemotherapy (n=117)						
All cases	440.3	440.5	441.1	441.5	441.0	443.9
CLL	243.1	243.1	243.7	244.2	244.2	246.4
non-CLL	197.1	196.7	197.5	197.3	196.8	196.9

Supplemental Material, Table S2: Excess relative risk per Gy (ERR/Gy) with 95% confidence interval (CI) for leukemia within categories of various factors. In contrast to Table 2, cases with direct interviews less than 2 years from start of chemotherapy are included.

Model Description	N cases	ERR/Gy (95% CI)	*P* value[a]	*P* interaction[b]
All cases	137	1.26 (0.03, 3.58)	0.041	
Leukemia subtype				
non-CLL	58	1.87 (-0.02, 6.54)	0.055	0.536
CLL	79	0.76 (<-0.38, 3.84)	0.352	
Proxy status[c]				
Proxy	69	5.10 (<-0.81, 29.29)		0.098
Direct interview	68	-0.10 (<-0.38, 1.74)		
0-1 years from start of chemotherapy	20	-0.47 (<-0.47, 1.02)	0.244	0.103
2-15 years from start of chemotherapy	48	1.45 (<-0.74, 7.62)	0.254	
Year of case diagnosis				
1986-1994	33	6.70 (0.27, 27.10)		0.040[d]
1995-2000	40	2.11 (-0.11, 8.87)		
2001-2006	64	0.26 (<-0.47, 2.47)		
Type of work performed in the 30-km Chornobyl zone				
Early responders	36	0.74 (-0.18, 3.01)		0.682
Military personnel	49	3.38 (-0.19, 10.54)		
Professional nuclear power workers	5	1.75 (<-0.54, 15.95)		
Other	47	2.02 (-0.69, 9.42)		
Time since first exposure, years				
0-9	38	5.02 (-0.04, 18.85)		0.048[d]
10-14	38	3.24 (0.20, 10.74)		
15-21	61	0.06 (<-0.47, 2.00)		
Age at first exposure, years				
20-34	31	0.43 (<-0.98, 6.24)		0.105[d]
35-41	36	0.58 (-0.58, 4.85)		
42-49	40	2.20 (-0.09, 9.84)		
50-63	30	1.87 (<-0.38, 9.49)		

[a] *P* value of departure of ERR/Gy from zero.

[b] *P* value for interaction effects.

[c] Background rate adjusted for proxy status.

[d] *P* value from the linear trend test.
